# Poly[[(1,10-phenanthroline){μ_3_-2,2′,2′′-[1,3,5-triazine-2,4,6-triyltris(sulfane­diyl)]triacetato}­cadmium] 0.42-hydrate]

**DOI:** 10.1107/S1600536811019210

**Published:** 2011-06-04

**Authors:** Chun-Jing Chi, Yan-Qiang Peng, Su-Yuan Zeng, De-Zhi Sun

**Affiliations:** a College of Chemistry and Chemical Engineering, Liaocheng University, Shandong 252059, People’s Republic of China

## Abstract

The asymmetric unit of the title complex, {[Cd(C_9_H_7_N_3_O_6_S_3_)(C_12_H_8_N_2_)]·0.42H_2_O}_*n*_, contains a Cd^II^ atom, one doubly deprotonated 2,2′,2′′-[1,3,5-triazine-2,4,6-triyltris(sulfanediyl)]triacetic acid ligand (HTTTA^2−^), a 1,10-phenanthroline (phen) ligand and a fractionally occupied water mol­ecule [site occupancy = 0.421 (15)]. The Cd^II^ atom is six-coordinated within a distorted octa­hedral coordination geometry. Six coordination arises from four O atoms derived from three different HTTTA^2−^ ligands, and two N atoms of the chelating phen mol­ecule. The incompletely deprotonated triazine ligand adopts a μ_3_-η^1^:η^1^:η^2^ coordination mode, resulting in the formation of chains along the *c* axis based on Cd_2_O_2_ dimeric units. Adjacent chains are stacked through π–π stacking [3.533 (2) Å between phen and triazine rings] and C—H⋯O inter­actions, forming supra­molecular sheets in the *ab* plane. Intra-and intermolecular O—H⋯O hydrogen bonds are also observed.

## Related literature

For background to metal-organic frameworks, see: Rao *et al.* (2004 ▶); Rowsell & Yaghi (2005 ▶); Wu *et al.* (2009 ▶). For similar structures, see: Lu *et al.* (2010 ▶); Wang *et al.* (2007 ▶).
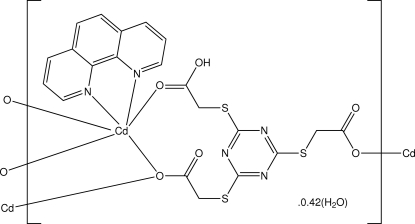


## Experimental

### 

#### Crystal data

[Cd(C_9_H_7_N_3_O_6_S_3_)(C_12_H_8_N_2_)]·0.42H_2_O
*M* *_r_* = 649.53
Triclinic, 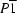

*a* = 10.618 (2) Å
*b* = 10.987 (2) Å
*c* = 12.601 (2) Å
α = 95.815 (3)°
β = 114.197 (2)°
γ = 113.909 (2)°
*V* = 1161.1 (4) Å^3^
*Z* = 2
Mo *K*α radiation
μ = 1.26 mm^−1^
*T* = 298 K
0.30 × 0.28 × 0.26 mm

#### Data collection

Bruker APEX CCD area-detector diffractometer
Absorption correction: multi-scan (*SADABS*; Sheldrick, 1996 ▶) *T*_min_ = 0.691, *T*_max_ = 0.720
6114 measured reflections
4024 independent reflections
3322 reflections with *I* > 2σ(*I*)
*R*_int_ = 0.018

#### Refinement

*R*[*F* ^2^ > 2σ(*F* ^2^)] = 0.030
*wR*(*F* ^2^) = 0.071
*S* = 1.07
4024 reflections
343 parameters
3 restraints
H atoms treated by a mixture of independent and constrained refinement
Δρ_max_ = 0.44 e Å^−3^
Δρ_min_ = −0.47 e Å^−3^

### 

Data collection: *SMART* (Bruker, 2000 ▶); cell refinement: *SAINT-Plus* (Bruker, 2000 ▶); data reduction: *SAINT-Plus*; program(s) used to solve structure: *SHELXS97* (Sheldrick, 2008 ▶); program(s) used to refine structure: *SHELXL97* (Sheldrick, 2008 ▶); molecular graphics: *SHELXTL* (Sheldrick, 2008 ▶); software used to prepare material for publication: *SHELXTL*.

## Supplementary Material

Crystal structure: contains datablock(s) I, global. DOI: 10.1107/S1600536811019210/tk2743sup1.cif

Structure factors: contains datablock(s) I. DOI: 10.1107/S1600536811019210/tk2743Isup2.hkl

Additional supplementary materials:  crystallographic information; 3D view; checkCIF report

## Figures and Tables

**Table 1:** Selected bond lengths (Å)

Cd1—O1	2.447 (2)
Cd1—O1^i^	2.274 (2)
Cd1—O4^i^	2.490 (3)
Cd1—O5^ii^	2.295 (2)
Cd1—N4	2.331 (3)
Cd1—N5	2.320 (3)

Symmetry codes: (i) 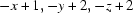; (ii) 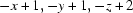.

**Table 2:** Hydrogen-bond geometry (Å, °)

*D*—H⋯*A*	*D*—H	H⋯*A*	*D*⋯*A*	*D*—H⋯*A*
O3—H3⋯O6^iii^	0.82	1.68	2.439 (4)	154
O7—H71⋯O2^iv^	0.75 (2)	2.35 (12)	2.984 (11)	142 (18)
C15—H15⋯O2^v^	0.93	2.50	3.294 (6)	143
C17—H17⋯O2^v^	0.93	2.57	3.353 (6)	142

Symmetry codes: (iii) 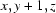; (iv) 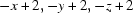; (v) 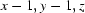.

**Fig. 1.**
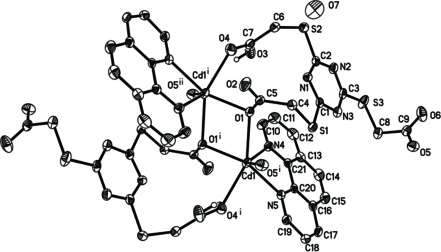
A view of the asymmetric unit of the title complex extended to show i) the coordination geometry about the Cd1 atom and ii) the coordinating mode of the µ3-ligand. The figure shows atom labels and 30% probability displacement ellipsoids for non-hydrogen atoms. Only the H3 atom is shown with the others omitted for clarity. Symmetry codes: (i) 2 - x, 1 - y, 1 - z and (ii) x, 1/2 - y, 1/2 + z.

**Fig. 2.**
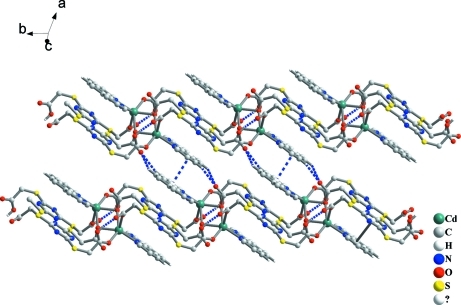
The two-dimensional sheet in the title complex connected by C—H···O and π-π stacking interactions (dashed blue lines). Hydrogen atoms are omitted for clarity.

## supplementary crystallographic information

### Comment

The assembly of coordination architectures has attracted much attention in recent years due to their potential applications in separation, sorption, hydrogen storage, and catalysis, as well as due to their intriguing topologies such as molecular ladders, grids, rings, boxes, honeycombs, and diamondoids (Rowsell & Yaghi, 2005). Flexible multi-functional carboxylic acids are widely investigated in this regard (Rao *et al.*, 2004; Rowsell & Yaghi 2005; Wu *et al.*, 2009). Previous reports of an alkaline earth and a series of lanthanide coordination complexes based on H_3_TTTA, 2,2',2''-[1,3,5-triazine-2,4,6-triyltris(sulfanediyl)]tris-acetic acid, have appeared (Lu, *et al.*, 2010; Wang, *et al.*, 2007). Herein, we obtained a new Cd^II^ complex assembled from this flexible ligand.

As shown in Fig.1, the asymmetric unit consists of one Cd^II^ ion, one HTTTA^2-^ dianion, a chelating 1,10-phenanthroline (phen) ligand and approximately half a disordered water molecule (site occupancy = 0.421 (15)). The Cd center is six-coordinated defined by four oxygen atoms derived from three different HTTTA^2-^ anions, and two nitrogen atoms of a chelating phen molecule; Table 1. The N5—Cd1—N4 angle is acute at 71.86 (9)° and consequently, the coordination geometry around the metal center is much distorted. The HTTTA^2-^ ligands act as µ_3_-bridges, connecting neighboring Cd centers to generate 1-D chains along the *c* axis. The H atom of the carboxylic group of the HTTTA^2-^ ligand was assigned to O3 according to the long C7—O3 distance of 1.283 (4) Å as well as O3—H3···O6 hydrogen bonding interactions, Table 2. Within the chains, Cd_2_O_2_ units are formed through the *η^2^*-bridged carboxylate oxygen atoms O1, with the Cd1···Cd1^i^ distance and Cd1—O1—Cd1^i^ (symmetry code: *i*, 1 - *x*, 2 - *y*, 2 - *z*.) angle being 3.829 (3) Å and 108.3 (3) °, respectively.

Neighboring chains are connected to each other through weak intermolecular π–π stacking interactions between phen and triazine rings with the average interplanar separation of 3.533 (2) Å. As a result, two-dimensional supramolecular sheets are formed along the *ab* plane, Fig. 2. These sheets are reinforced *via* nonclassical weak C—H···O interactions Table 2.

### Experimental

A mixture of 2,2',2''-((1,3,5-triazine-2,4,6-triyl)tris(sulfanediyl))triacetic acid (0.010 g, 0.025 mmol), phenanthroline (0.008 g, 0.05 mmol) and Cd(OAc)_2_.6H_2_O (0.013 g, 0.025 mmol) in 10 mL H_2_O was placed in a Parr Teflon-lined stainless steel vessel and heated to 80 °C for 24 h. The reaction system was cooled to room temperature slowly and yellow blocks were obtained. After filtration, the crystals were washed with water and dried in air. (Yield 64% based on Cd(OAc)_2_.6H_2_O). Calcd.: C 38.80, H 2.44, N 10.78; C_21_H_15.84_CdN_5_O_6.42_S_3_ requires: C 38.43, H 2.70, N 10.42 %. IR (KBr pellet): 3421 (m,br), 2908 (w), 1591 (m), 1517 (vw), 1425 (m), 1381 (m), 1266 (m), 1246 (m), 1222 (m), 855 (m), 785 (w), 730 (m), 669 (w) cm^-1^.

### Refinement

The O7 water molecule was fractionally disordered and was refined isotropically to an occupancy of 0.421 (15). The H atoms on this water molecule were located from a difference Fourier Map. The O—H bond distances were fixed to 0.75 (2) Å, and the H—O7—H angle was fixed to 109.79 (4) °; only one of the H atoms was found to be engaged in hydrogen bonding interactions. The remaining H-atoms were positioned geometrically and constrained to ride on their parent atoms with C—H = 0.93 - 0.97 Å and O—H = 0.82 (2) Å, and with *U*_iso_(H) = 1.2*U*_eq_(C,O).

### Crystal data

**Table:**

[Cd(C_9_H_7_N_3_O_6_S_3_)(C_12_H_8_N_2_)]·0.42H_2_O	*Z* = 2
*M**_r_* = 649.53	*F*(000) = 648
Triclinic, *P*1	*D*_x_ = 1.858 Mg m^−^^3^
Hall symbol: -P 1	Mo *K*α radiation, λ = 0.71073 Å
*a* = 10.618 (2) Å	Cell parameters from 3004 reflections
*b* = 10.987 (2) Å	θ = 2.3–27.8°
*c* = 12.601 (2) Å	µ = 1.26 mm^−^^1^
α = 95.815 (3)°	*T* = 298 K
β = 114.197 (2)°	Block, yellow
γ = 113.909 (2)°	0.30 × 0.28 × 0.26 mm
*V* = 1161.1 (4) Å^3^	

### Data collection

**Table:**

Bruker APEX CCD area-detector diffractometer	4024 independent reflections
Radiation source: fine-focus sealed tube	3322 reflections with *I* > 2σ(*I*)
graphite	*R*_int_ = 0.018
φ and ω scans	θ_max_ = 25.0°, θ_min_ = 1.9°
Absorption correction: multi-scan (*SADABS*; Sheldrick, 1996)	*h* = −12→6
*T*_min_ = 0.691, *T*_max_ = 0.720	*k* = −11→13
6114 measured reflections	*l* = −14→14

### Refinement

**Table:**

Refinement on *F*^2^	Primary atom site location: structure-invariant direct methods
Least-squares matrix: full	Secondary atom site location: difference Fourier map
*R*[*F*^2^ > 2σ(*F*^2^)] = 0.030	Hydrogen site location: inferred from neighbouring sites
*wR*(*F*^2^) = 0.071	H atoms treated by a mixture of independent and constrained refinement
*S* = 1.07	*w* = 1/[σ^2^(*F*_o_^2^) + (0.0324*P*)^2^] where *P* = (*F*_o_^2^ + 2*F*_c_^2^)/3
4024 reflections	(Δ/σ)_max_ = 0.001
343 parameters	Δρ_max_ = 0.44 e Å^−^^3^
3 restraints	Δρ_min_ = −0.47 e Å^−^^3^

### Special details

**Table:**

Geometry. All e.s.d.'s (except the e.s.d. in the dihedral angle between two l.s. planes) are estimated using the full covariance matrix. The cell e.s.d.'s are taken into account individually in the estimation of e.s.d.'s in distances, angles and torsion angles; correlations between e.s.d.'s in cell parameters are only used when they are defined by crystal symmetry. An approximate (isotropic) treatment of cell e.s.d.'s is used for estimating e.s.d.'s involving l.s. planes.
Refinement. Refinement of *F*^2^ against ALL reflections. The weighted *R*-factor *wR* and goodness of fit *S* are based on *F*^2^, conventional *R*-factors *R* are based on *F*, with *F* set to zero for negative *F*^2^. The threshold expression of *F*^2^ > σ(*F*^2^) is used only for calculating *R*-factors(gt) *etc*. and is not relevant to the choice of reflections for refinement. *R*-factors based on *F*^2^ are statistically about twice as large as those based on *F*, and *R*- factors based on ALL data will be even larger.

### Fractional atomic coordinates and isotropic or equivalent isotropic displacement parameters (Å^2^)

**Table:**

	*x*	*y*	*z*	*U*_iso_*/*U*_eq_	Occ. (<1)
Cd1	0.42035 (3)	0.85652 (2)	1.06635 (2)	0.03328 (10)	
C1	0.4810 (4)	0.6039 (3)	0.8043 (3)	0.0323 (8)	
C2	0.4472 (4)	0.6269 (3)	0.6212 (3)	0.0359 (8)	
C3	0.3023 (4)	0.4203 (3)	0.6345 (3)	0.0356 (8)	
C4	0.7363 (4)	0.8372 (3)	0.9962 (3)	0.0331 (8)	
H4A	0.8205	0.8656	1.0798	0.040*	
H4B	0.7782	0.8275	0.9424	0.040*	
C5	0.6972 (4)	0.9555 (3)	0.9826 (3)	0.0346 (8)	
C6	0.6109 (4)	0.8984 (3)	0.6309 (3)	0.0426 (9)	
H6A	0.6908	0.9023	0.7073	0.051*	
H6B	0.6661	0.9586	0.5955	0.051*	
C7	0.5189 (4)	0.9579 (4)	0.6602 (3)	0.0385 (8)	
C8	0.1498 (4)	0.1658 (4)	0.6678 (3)	0.0418 (9)	
H8A	0.1936	0.2377	0.7438	0.050*	
H8B	0.0417	0.0990	0.6446	0.050*	
C9	0.2466 (4)	0.0902 (3)	0.6898 (3)	0.0362 (8)	
C10	0.1914 (4)	0.7138 (4)	0.7677 (3)	0.0456 (9)	
H10	0.2475	0.8042	0.7672	0.055*	
C11	0.0686 (5)	0.6091 (5)	0.6563 (3)	0.0555 (11)	
H11	0.0439	0.6302	0.5833	0.067*	
C12	−0.0148 (4)	0.4762 (4)	0.6546 (3)	0.0501 (10)	
H12	−0.0969	0.4063	0.5806	0.060*	
C13	0.0238 (4)	0.4452 (4)	0.7654 (3)	0.0374 (8)	
C14	−0.0603 (4)	0.3101 (4)	0.7722 (4)	0.0484 (10)	
H14	−0.1414	0.2364	0.7003	0.058*	
C15	−0.0245 (4)	0.2871 (4)	0.8809 (4)	0.0458 (9)	
H15	−0.0817	0.1981	0.8832	0.055*	
C16	0.1006 (4)	0.3981 (3)	0.9934 (3)	0.0350 (8)	
C17	0.1391 (4)	0.3790 (4)	1.1094 (4)	0.0418 (9)	
H17	0.0825	0.2921	1.1153	0.050*	
C18	0.2594 (4)	0.4883 (4)	1.2119 (3)	0.0415 (9)	
H18	0.2871	0.4769	1.2890	0.050*	
C19	0.3412 (4)	0.6180 (4)	1.2008 (3)	0.0385 (8)	
H19	0.4236	0.6922	1.2720	0.046*	
C20	0.1881 (3)	0.5322 (3)	0.9908 (3)	0.0278 (7)	
C21	0.1479 (4)	0.5563 (3)	0.8738 (3)	0.0296 (7)	
N1	0.5244 (3)	0.6901 (3)	0.7429 (2)	0.0339 (7)	
N2	0.3350 (4)	0.4923 (3)	0.5603 (3)	0.0425 (7)	
N3	0.3688 (3)	0.4680 (3)	0.7551 (2)	0.0365 (7)	
N4	0.2304 (3)	0.6890 (3)	0.8734 (2)	0.0344 (6)	
N5	0.3072 (3)	0.6407 (3)	1.0937 (2)	0.0311 (6)	
O1	0.5578 (3)	0.9325 (2)	0.9501 (2)	0.0360 (5)	
O2	0.8069 (3)	1.0707 (3)	1.0042 (3)	0.0579 (7)	
O3	0.3680 (3)	0.8904 (3)	0.5892 (2)	0.0601 (8)	
H3	0.3281	0.9298	0.6119	0.072*	
O4	0.5906 (3)	1.0653 (3)	0.7466 (2)	0.0525 (7)	
O5	0.3310 (3)	0.1012 (3)	0.7971 (2)	0.0483 (6)	
O6	0.2284 (3)	0.0185 (3)	0.5955 (2)	0.0665 (8)	
S1	0.57763 (10)	0.66671 (9)	0.96406 (7)	0.0340 (2)	
S2	0.49678 (13)	0.72178 (10)	0.52847 (9)	0.0503 (3)	
S3	0.14725 (13)	0.24605 (10)	0.55044 (9)	0.0552 (3)	
O7	0.9389 (12)	0.8880 (13)	0.7511 (12)	0.098 (5)	0.421 (15)
H71	0.97 (2)	0.876 (19)	0.813 (8)	0.17 (10)*	0.421 (15)
H72	0.95 (3)	0.85 (2)	0.711 (15)	0.3 (2)*	0.421 (15)

### Atomic displacement parameters (Å^2^)

**Table:**

	*U*^11^	*U*^22^	*U*^33^	*U*^12^	*U*^13^	*U*^23^
Cd1	0.03706 (16)	0.02399 (14)	0.03189 (15)	0.01304 (11)	0.01291 (11)	0.01064 (10)
C1	0.0352 (19)	0.0324 (19)	0.0380 (19)	0.0237 (16)	0.0181 (15)	0.0130 (15)
C2	0.047 (2)	0.035 (2)	0.037 (2)	0.0277 (18)	0.0229 (17)	0.0142 (16)
C3	0.040 (2)	0.0288 (18)	0.037 (2)	0.0221 (16)	0.0128 (16)	0.0106 (15)
C4	0.0322 (18)	0.0341 (19)	0.0313 (18)	0.0170 (15)	0.0137 (15)	0.0098 (15)
C5	0.047 (2)	0.0301 (19)	0.0312 (18)	0.0199 (18)	0.0213 (16)	0.0127 (15)
C6	0.052 (2)	0.034 (2)	0.047 (2)	0.0192 (18)	0.0299 (18)	0.0166 (17)
C7	0.047 (2)	0.0312 (19)	0.0337 (19)	0.0173 (18)	0.0175 (17)	0.0140 (16)
C8	0.043 (2)	0.033 (2)	0.043 (2)	0.0192 (17)	0.0150 (17)	0.0107 (16)
C9	0.0319 (19)	0.0278 (18)	0.035 (2)	0.0100 (15)	0.0105 (16)	0.0074 (15)
C10	0.050 (2)	0.046 (2)	0.039 (2)	0.0238 (19)	0.0170 (18)	0.0214 (18)
C11	0.056 (3)	0.071 (3)	0.034 (2)	0.036 (2)	0.0135 (19)	0.019 (2)
C12	0.036 (2)	0.057 (3)	0.032 (2)	0.019 (2)	0.0042 (16)	−0.0011 (18)
C13	0.0286 (18)	0.041 (2)	0.0357 (19)	0.0166 (16)	0.0120 (15)	0.0046 (16)
C14	0.031 (2)	0.033 (2)	0.051 (2)	0.0029 (17)	0.0125 (17)	−0.0060 (17)
C15	0.034 (2)	0.028 (2)	0.062 (3)	0.0072 (16)	0.0226 (18)	0.0067 (18)
C16	0.0305 (18)	0.0285 (18)	0.052 (2)	0.0161 (15)	0.0241 (16)	0.0120 (16)
C17	0.049 (2)	0.0315 (19)	0.070 (3)	0.0250 (18)	0.043 (2)	0.0268 (19)
C18	0.056 (2)	0.044 (2)	0.043 (2)	0.031 (2)	0.0327 (19)	0.0223 (18)
C19	0.050 (2)	0.034 (2)	0.0316 (19)	0.0229 (18)	0.0183 (17)	0.0114 (15)
C20	0.0267 (17)	0.0246 (17)	0.0386 (19)	0.0158 (14)	0.0185 (15)	0.0098 (14)
C21	0.0256 (17)	0.0306 (18)	0.0361 (18)	0.0160 (15)	0.0161 (14)	0.0099 (15)
N1	0.0421 (17)	0.0325 (16)	0.0294 (15)	0.0190 (14)	0.0186 (13)	0.0116 (13)
N2	0.057 (2)	0.0333 (17)	0.0352 (16)	0.0241 (15)	0.0195 (15)	0.0104 (13)
N3	0.0386 (16)	0.0305 (16)	0.0365 (17)	0.0182 (14)	0.0144 (13)	0.0100 (13)
N4	0.0360 (16)	0.0339 (16)	0.0315 (15)	0.0178 (13)	0.0142 (13)	0.0127 (13)
N5	0.0327 (15)	0.0268 (15)	0.0326 (15)	0.0152 (13)	0.0150 (12)	0.0085 (12)
O1	0.0432 (14)	0.0350 (13)	0.0465 (14)	0.0261 (12)	0.0274 (12)	0.0215 (11)
O2	0.0495 (17)	0.0328 (15)	0.084 (2)	0.0135 (13)	0.0330 (15)	0.0213 (14)
O3	0.0507 (17)	0.0477 (16)	0.0560 (17)	0.0255 (14)	0.0089 (14)	−0.0075 (13)
O4	0.0543 (16)	0.0355 (14)	0.0496 (16)	0.0102 (13)	0.0257 (13)	−0.0012 (12)
O5	0.0434 (15)	0.0679 (18)	0.0361 (14)	0.0318 (14)	0.0164 (12)	0.0213 (13)
O6	0.078 (2)	0.075 (2)	0.0409 (16)	0.0550 (18)	0.0101 (14)	0.0029 (15)
S1	0.0398 (5)	0.0301 (5)	0.0326 (5)	0.0182 (4)	0.0167 (4)	0.0131 (4)
S2	0.0826 (8)	0.0418 (5)	0.0415 (5)	0.0332 (5)	0.0399 (5)	0.0174 (4)
S3	0.0654 (7)	0.0296 (5)	0.0379 (5)	0.0169 (5)	0.0052 (5)	0.0089 (4)
O7	0.076 (6)	0.102 (8)	0.076 (8)	0.033 (5)	0.014 (5)	0.025 (6)

### Geometric parameters (Å, °)

**Table:**

Cd1—O1	2.447 (2)	C9—O6	1.253 (4)
Cd1—O1^i^	2.274 (2)	C10—N4	1.316 (4)
Cd1—O4^i^	2.490 (3)	C10—C11	1.397 (5)
Cd1—O5^ii^	2.295 (2)	C10—H10	0.9300
Cd1—N4	2.331 (3)	C11—C12	1.359 (5)
Cd1—N5	2.320 (3)	C11—H11	0.9300
C1—N1	1.340 (4)	C12—C13	1.405 (5)
C1—N3	1.341 (4)	C12—H12	0.9300
C1—S1	1.742 (3)	C13—C21	1.405 (4)
C2—N2	1.336 (4)	C13—C14	1.422 (5)
C2—N1	1.341 (4)	C14—C15	1.344 (5)
C2—S2	1.742 (3)	C14—H14	0.9300
C3—N3	1.320 (4)	C15—C16	1.433 (5)
C3—N2	1.348 (4)	C15—H15	0.9300
C3—S3	1.761 (3)	C16—C20	1.400 (4)
C4—C5	1.522 (4)	C16—C17	1.410 (5)
C4—S1	1.800 (3)	C17—C18	1.354 (5)
C4—H4A	0.9700	C17—H17	0.9300
C4—H4B	0.9700	C18—C19	1.393 (5)
C5—O2	1.228 (4)	C18—H18	0.9300
C5—O1	1.265 (4)	C19—N5	1.325 (4)
C6—C7	1.512 (5)	C19—H19	0.9300
C6—S2	1.790 (4)	C20—N5	1.349 (4)
C6—H6A	0.9700	C20—C21	1.443 (4)
C6—H6B	0.9700	C21—N4	1.365 (4)
C7—O4	1.220 (4)	O1—Cd1^i^	2.274 (2)
C7—O3	1.283 (4)	O3—H3	0.8201
C8—C9	1.526 (5)	O4—Cd1^i^	2.490 (3)
C8—S3	1.793 (4)	O5—Cd1^ii^	2.295 (2)
C8—H8A	0.9700	O7—H71	0.75 (2)
C8—H8B	0.9700	O7—H72	0.75 (2)
C9—O5	1.237 (4)		
			
O1^i^—Cd1—O5^ii^	107.00 (9)	C11—C10—H10	118.9
O1^i^—Cd1—N5	154.48 (9)	C12—C11—C10	120.0 (3)
O5^ii^—Cd1—N5	90.50 (9)	C12—C11—H11	120.0
O1^i^—Cd1—N4	107.24 (9)	C10—C11—H11	120.0
O5^ii^—Cd1—N4	131.23 (10)	C11—C12—C13	119.5 (3)
N5—Cd1—N4	71.86 (9)	C11—C12—H12	120.2
O1^i^—Cd1—O1	71.65 (9)	C13—C12—H12	120.2
O5^ii^—Cd1—O1	79.40 (8)	C12—C13—C21	117.2 (3)
N5—Cd1—O1	131.20 (8)	C12—C13—C14	123.2 (3)
N4—Cd1—O1	79.61 (9)	C21—C13—C14	119.6 (3)
O1^i^—Cd1—O4^i^	82.53 (8)	C15—C14—C13	121.1 (3)
O5^ii^—Cd1—O4^i^	84.79 (9)	C15—C14—H14	119.5
N5—Cd1—O4^i^	80.69 (9)	C13—C14—H14	119.5
N4—Cd1—O4^i^	133.26 (9)	C14—C15—C16	120.8 (3)
O1—Cd1—O4^i^	143.94 (8)	C14—C15—H15	119.6
N1—C1—N3	126.4 (3)	C16—C15—H15	119.6
N1—C1—S1	119.5 (2)	C20—C16—C17	117.5 (3)
N3—C1—S1	114.1 (2)	C20—C16—C15	120.0 (3)
N2—C2—N1	126.4 (3)	C17—C16—C15	122.5 (3)
N2—C2—S2	114.1 (2)	C18—C17—C16	119.4 (3)
N1—C2—S2	119.4 (2)	C18—C17—H17	120.3
N3—C3—N2	127.3 (3)	C16—C17—H17	120.3
N3—C3—S3	121.0 (3)	C17—C18—C19	119.3 (3)
N2—C3—S3	111.6 (2)	C17—C18—H18	120.4
C5—C4—S1	117.4 (2)	C19—C18—H18	120.4
C5—C4—H4A	108.0	N5—C19—C18	123.1 (3)
S1—C4—H4A	108.0	N5—C19—H19	118.4
C5—C4—H4B	108.0	C18—C19—H19	118.4
S1—C4—H4B	108.0	N5—C20—C16	122.6 (3)
H4A—C4—H4B	107.2	N5—C20—C21	118.6 (3)
O2—C5—O1	123.5 (3)	C16—C20—C21	118.8 (3)
O2—C5—C4	116.3 (3)	N4—C21—C13	122.4 (3)
O1—C5—C4	120.2 (3)	N4—C21—C20	117.9 (3)
C7—C6—S2	116.0 (3)	C13—C21—C20	119.7 (3)
C7—C6—H6A	108.3	C1—N1—C2	113.5 (3)
S2—C6—H6A	108.3	C2—N2—C3	113.0 (3)
C7—C6—H6B	108.3	C3—N3—C1	113.3 (3)
S2—C6—H6B	108.3	C10—N4—C21	118.7 (3)
H6A—C6—H6B	107.4	C10—N4—Cd1	125.7 (2)
O4—C7—O3	124.8 (4)	C21—N4—Cd1	115.45 (19)
O4—C7—C6	119.2 (3)	C19—N5—C20	118.0 (3)
O3—C7—C6	116.0 (3)	C19—N5—Cd1	125.7 (2)
C9—C8—S3	113.0 (3)	C20—N5—Cd1	116.0 (2)
C9—C8—H8A	109.0	C5—O1—Cd1^i^	101.51 (19)
S3—C8—H8A	109.0	C5—O1—Cd1	124.8 (2)
C9—C8—H8B	109.0	Cd1^i^—O1—Cd1	108.35 (9)
S3—C8—H8B	109.0	C7—O3—H3	109.3
H8A—C8—H8B	107.8	C7—O4—Cd1^i^	136.9 (2)
O5—C9—O6	126.1 (3)	C9—O5—Cd1^ii^	143.1 (2)
O5—C9—C8	118.0 (3)	C1—S1—C4	101.33 (16)
O6—C9—C8	115.9 (3)	C2—S2—C6	101.51 (17)
N4—C10—C11	122.2 (3)	C3—S3—C8	103.00 (16)
N4—C10—H10	118.9	H71—O7—H72	110 (6)
			
S1—C4—C5—O2	−179.5 (3)	N5—Cd1—N4—C10	178.2 (3)
S1—C4—C5—O1	0.1 (4)	O1—Cd1—N4—C10	−41.9 (3)
S2—C6—C7—O4	165.6 (3)	O4^i^—Cd1—N4—C10	121.1 (3)
S2—C6—C7—O3	−15.3 (4)	O1^i^—Cd1—N4—C21	−150.7 (2)
S3—C8—C9—O5	139.6 (3)	O5^ii^—Cd1—N4—C21	76.5 (2)
S3—C8—C9—O6	−42.5 (4)	N5—Cd1—N4—C21	2.5 (2)
N4—C10—C11—C12	−0.2 (6)	O1—Cd1—N4—C21	142.4 (2)
C10—C11—C12—C13	−0.2 (6)	O4^i^—Cd1—N4—C21	−54.7 (3)
C11—C12—C13—C21	0.7 (5)	C18—C19—N5—C20	−0.4 (5)
C11—C12—C13—C14	178.1 (4)	C18—C19—N5—Cd1	174.1 (2)
C12—C13—C14—C15	−176.4 (4)	C16—C20—N5—C19	−0.2 (5)
C21—C13—C14—C15	0.9 (6)	C21—C20—N5—C19	178.8 (3)
C13—C14—C15—C16	−0.5 (6)	C16—C20—N5—Cd1	−175.2 (2)
C14—C15—C16—C20	−0.4 (5)	C21—C20—N5—Cd1	3.8 (4)
C14—C15—C16—C17	178.4 (4)	O1^i^—Cd1—N5—C19	−85.7 (3)
C20—C16—C17—C18	−1.4 (5)	O5^ii^—Cd1—N5—C19	48.4 (3)
C15—C16—C17—C18	179.7 (3)	N4—Cd1—N5—C19	−177.9 (3)
C16—C17—C18—C19	0.9 (5)	O1—Cd1—N5—C19	124.7 (3)
C17—C18—C19—N5	0.0 (5)	O4^i^—Cd1—N5—C19	−36.2 (3)
C17—C16—C20—N5	1.1 (5)	O1^i^—Cd1—N5—C20	88.9 (3)
C15—C16—C20—N5	−180.0 (3)	O5^ii^—Cd1—N5—C20	−137.0 (2)
C17—C16—C20—C21	−177.9 (3)	N4—Cd1—N5—C20	−3.3 (2)
C15—C16—C20—C21	1.0 (5)	O1—Cd1—N5—C20	−60.7 (2)
C12—C13—C21—N4	−0.9 (5)	O4^i^—Cd1—N5—C20	138.4 (2)
C14—C13—C21—N4	−178.4 (3)	O2—C5—O1—Cd1^i^	4.0 (4)
C12—C13—C21—C20	177.2 (3)	C4—C5—O1—Cd1^i^	−175.6 (2)
C14—C13—C21—C20	−0.3 (5)	O2—C5—O1—Cd1	−118.2 (3)
N5—C20—C21—N4	−1.5 (4)	C4—C5—O1—Cd1	62.2 (4)
C16—C20—C21—N4	177.5 (3)	O1^i^—Cd1—O1—C5	119.1 (3)
N5—C20—C21—C13	−179.7 (3)	O5^ii^—Cd1—O1—C5	7.1 (2)
C16—C20—C21—C13	−0.6 (5)	N5—Cd1—O1—C5	−74.2 (3)
N3—C1—N1—C2	−2.2 (5)	N4—Cd1—O1—C5	−128.6 (3)
S1—C1—N1—C2	176.4 (2)	O4^i^—Cd1—O1—C5	72.6 (3)
N2—C2—N1—C1	1.0 (5)	O5^ii^—Cd1—O1—Cd1^i^	−112.05 (11)
S2—C2—N1—C1	−176.7 (2)	N5—Cd1—O1—Cd1^i^	166.69 (9)
N1—C2—N2—C3	0.3 (5)	N4—Cd1—O1—Cd1^i^	112.23 (11)
S2—C2—N2—C3	178.2 (2)	O4^i^—Cd1—O1—Cd1^i^	−46.50 (17)
N3—C3—N2—C2	−0.8 (5)	O3—C7—O4—Cd1^i^	54.0 (5)
S3—C3—N2—C2	177.1 (2)	C6—C7—O4—Cd1^i^	−127.0 (3)
N2—C3—N3—C1	−0.2 (5)	O6—C9—O5—Cd1^ii^	48.3 (6)
S3—C3—N3—C1	−177.8 (2)	C8—C9—O5—Cd1^ii^	−134.1 (3)
N1—C1—N3—C3	1.8 (5)	N1—C1—S1—C4	−7.9 (3)
S1—C1—N3—C3	−176.8 (2)	N3—C1—S1—C4	170.9 (2)
C11—C10—N4—C21	0.0 (6)	C5—C4—S1—C1	80.6 (3)
C11—C10—N4—Cd1	−175.6 (3)	N2—C2—S2—C6	167.5 (3)
C13—C21—N4—C10	0.5 (5)	N1—C2—S2—C6	−14.5 (3)
C20—C21—N4—C10	−177.6 (3)	C7—C6—S2—C2	−71.7 (3)
C13—C21—N4—Cd1	176.6 (2)	N3—C3—S3—C8	−11.5 (3)
C20—C21—N4—Cd1	−1.5 (4)	N2—C3—S3—C8	170.4 (3)
O1^i^—Cd1—N4—C10	25.0 (3)	C9—C8—S3—C3	−95.0 (3)
O5^ii^—Cd1—N4—C10	−107.7 (3)		

Symmetry codes: (i) −*x*+1, −*y*+2, −*z*+2; (ii) −*x*+1, −*y*+1, −*z*+2.

### Hydrogen-bond geometry (Å, °)

**Table:**

*D*—H···*A*	*D*—H	H···*A*	*D*···*A*	*D*—H···*A*
O3—H3···O6^iii^	0.82	1.68	2.439 (4)	154
O7—H71···O2^iv^	0.75 (2)	2.35 (12)	2.984 (11)	142 (18)
C15—H15···O2^v^	0.93	2.50	3.294 (6)	143
C17—H17···O2^v^	0.93	2.57	3.353 (6)	142

Symmetry codes: (iii) *x*, *y*+1, *z*; (iv) −*x*+2, −*y*+2, −*z*+2; (v) *x*−1, *y*−1, *z*.
